# Therapeutics

**Published:** 1879-05

**Authors:** 


### THERAPEUTICS.

A NEW DISINFECTANT.—(*Lyon Médical.*)

Under this title Dr. John Day, of Australia, recommends (*Alger Médical*) for use in general hospitals and for destroying contagion germs, a mixture of one part of rectified essence of turpentine and seven parts of benzine, with five drops of essence of verbena to the ounce of the mixture. Its disinfectant properties are due to its capability of absorbing oxygen and converting it into ozone. Furniture, clothing, books, etc., can be disinfected with it without injury, and whether it be applied to a porous or non-absorbing surface, its action persists indefinitely. It can be applied with a sponge or brush, or an object be immersed in it. One can convince himself of its vigor of action at any time by dropping some solution of iodide of potassium on the substances thus treated; decomposition of the salt occurs in the presence of ozone, and the characteristics of free iodine are easily recognized.

OBSTINATE CASE OF HICCOUGH CURED BY THE PASSAGE OF AN ŒSOPHAGEAL SOUND. By Dr. E. Barré. (*L' Union Médicale*, March 17, 1879.)

V. B., employed in a bank. Age 45. Excitable temperament. Lost a little girl, January, 1876, from tubercular meningitis. Emotion brought on a continual and insupportable hiccough. Consulted a physician, and by the use of ether and bromide of potassium obtained relief, only to be again subject to the same annoyance. The ether, bromides and morphia no longer gave him relief. A second physician was consulted, with only a slight relief.

Wearied, he resorted to a herbalist; obtained no result. Then went to *l'Hôpital Lariboisière*. Followed directions faithfully, without relief.

His general health now became seriously affected by the continual disturbance.

On November 6, 1878, he went to consult Dr. Barré. He administered medicine, the chief ingredient of which was chloroform. This failed completely.

A general course of tonics was tried, with no success. On the 12th of January, the patient repeated his visit to Dr. B., and manifested greater discouragement than ever. The doctor confesses that he himself was not less discouraged. He tried compression, without effect. Just then his eyes alighted on an

œsophageal sound which lay upon his desk. Without much thought he passed the sound into the œsophagus; he does not say how far. Immediately his patient fainted. The sound was quickly withdrawn, and efforts directed to his revival. Dr. B. reproached himself for having resorted so thoughtlessly to such a measure, but was encouraged on the appearance of his patient the next day, with the assurance that he had only experienced three slight attacks since the former operation. The physician introduced the sound again, this time without any inconvenience, and at the time of writing, thirty-seven days after, no return of the hiccough had manifested itself.

A HIGHLY VASCULAR NÆVUS TREATED BY INTERSTITIAL INJECTION OF A 15 PER CENT. SOLUTION OF CANTHARIDINE IN CHLOROFORM.

On Sept. 21st, 1878, a child was brought to the Hôpital Saint Antoine, bearing in the inguinal region a prominent nævus of bright red color, the size of a large hazel nut, from which, from time to time, slight hæmorrhages took place, which led its mother to seek a cure of the little tumor. On the 22d there was injected half a gram of the solution, containing seven and a half miligrams of cantharidine; at the moment the child cried, but in half an hour was quiet. Two hours after it complained of smarting and pain in the tumor; three hours after a congestive zone around the nævus; twelve hours after a vesicle, and in twenty-four hours an eschar, which enlarged the following days; fourth day eschar very distinct, and several small vesicles in the healthy skin around the nævus. About the twelfth day eschar completely formed and, on the twentieth, commencement of line of separation on a level with healthy skin, for the eschar was not exactly limited to the tumor. One month after, eschar on the point of falling, and in two months there remained a slight wound, with tendency to heal; two and a half months after, the cicatrization was complete, with tendency to diminish; finally, three months after, there remained but a superficial scar, without any appearance of the vascular tumor.

CONCERNING PLUMBIC NITRATE.—(*Med. & Surg. Reporter.*)

Dr. Marsh, of Louisiana, some years ago called attention to the great value of plumbic nitrate in many skin affections and erosions. Recently, an Italian, Dr. Galletti, has added his testimony as to its merits. He states that he has cured three cases of epithelioma by dusting the powder over the affected part; recovery taking place after four or five applications. Two obstinate ulcers of the foot which had proved rebellious to other treatment quickly recovered after similar applications. Dr. Vanzetti has recently recommended the use of this salt, also, in onychia maligna.

CHLORAL AS A REVULSIVE.—(*Bulletin de Therap.*)

Dr. Peyraud states that chloral made into a mass with tragacanth, spread on paper or cloth, and applied to the skin, will produce a blister without pain. Applied as a powder on cotton it causes burning pain. By the former method a portion is absorbed and sleep produced. Its action is not quite as certain as that of cantharides, but as a mild revulsive and agreeable vesicant its action is very agreeable.

---

THE Annual Commencement of the Medical Department of the State University of Iowa, took place at Iowa City, on the evening of March 5th, 1879, at which fifteen candidates received the degree of Doctor of Medicine. The degrees were conferred by his excellency Gov. John H. Gear, President of the Board of Regents. The faculty address was delivered by the President of the University, J. L. Pickard. The class valedictory was delivered by Fred. H. Little, of Muscatine. This occasion closed the third year of operation in the department, of the "three years graduated course" of study, and a thorough revision of the plan of this course, with the addition of a preliminary examination of applicants for matriculation, are the steps in advance announced for next session.

